# Monitoring Perinatal Gut Microbiota in Mouse Models by Mass Spectrometry Approaches: Parental Genetic Background and Breastfeeding Effects

**DOI:** 10.3389/fmicb.2016.01523

**Published:** 2016-09-26

**Authors:** Stefano Levi Mortera, Federica Del Chierico, Pamela Vernocchi, Maria M. Rosado, Agnese Cavola, Marco Chierici, Luisa Pieroni, Andrea Urbani, Rita Carsetti, Isabella Lante, Bruno Dallapiccola, Lorenza Putignani

**Affiliations:** ^1^Human Microbiome Unit, Area of Genetic and Rare Diseases, Bambino Gesù Children’s Hospital, IRCCSRome, Italy; ^2^Immunology Research Area, B-cell Physiopathology Unit and Diagnostic Immunology Unit, Bambino Gesù Children’s Hospital, IRCCSRome, Italy; ^3^Department of Experimental Medicine, University of Rome Tor VergataRome, Italy; ^4^Fondazione Bruno KesslerTrento, Italy; ^5^IRCCS-Santa Lucia FoundationRome, Italy; ^6^Istituto di Biochimica e Biochimica Clinica, Università Cattolica del Sacro CuoreRome, Italy; ^7^Laboratory Medicine Department, San Camillo HospitalTreviso, Italy; ^8^Bambino Gesù Children’s Hospital, IRCCSRome, Italy; ^9^Unit of Parasitology, Department of Laboratory, Bambino Gesù Children’s Hospital, IRCCSRome, Italy

**Keywords:** mouse gut microbiota (MGM), MGM metaproteomics, MGM programming phylotypes, host genetics and maternal milk induced metaproteome, axenic culture-based MALDI–TOF MS

## Abstract

At birth, contact with external stimuli, such as nutrients derived from food, is necessary to modulate the symbiotic balance between commensal and pathogenic bacteria, protect against bacterial dysbiosis, and initiate the development of the mucosal immune response. Among a variety of different feeding patterns, breastfeeding represents the best modality. In fact, the capacity of breast milk to modulate the composition of infants’ gut microbiota leads to beneficial effects on their health. In this study, we used newborn mice as a model to evaluate the effect of parental genetic background (i.e., IgA-producing mice and IgA-deficient mice) and feeding modulation (i.e., maternal feeding and cross-feeding) on the onset and shaping of gut microbiota after birth. To investigate these topics, we used either a culturomic approach that employed Matrix Assisted Laser Desorption Ionization Time-of-Flight Mass Spectrometry (MS), or bottom–up Liquid Chromatography, with subsequent MSMS shotgun metaproteomic analysis that compared and assembled results of the two techniques. We found that the microbial community was enriched by lactic acid bacteria when pups were breastfed by wild-type (WT) mothers, while IgA-deficient milk led to an increase in the opportunistic bacterial pathogen (OBP) population. Cross-feeding results suggested that IgA supplementation promoted the exclusion of some OBPs and the temporary appearance of beneficial species in pups fed by WT foster mothers. Our results show that both techniques yield a picture of microbiota from different angles and with varying depths. In particular, our metaproteomic pipeline was found to be a reliable tool in the description of microbiota. Data from these studies are available via ProteomeXchange, with identifier PXD004033.

## Introduction

At birth, newborns rapidly come in contact with a number of pioneer bacteria, which immediately colonize skin, lung, and intestinal niches (Levy, 2007). Moreover, the influence of external stimuli, such as nutrients derived from food, is necessary to: (i) modulate the symbiotic balance between commensal and pathogenic bacteria (Putignani et al., 2014; Del Chierico et al., 2015); (ii) protect against bacterial dysbiosis; and (iii) initiate the development of a mucosal immune response (Alexander et al., 2014; Mirpuri et al., 2014). Of the variety of feeding patterns available, breastfeeding is believed to be the best. In particular, breast milk modulates the composition of infants’ gut microbiota, with consequent beneficial effects on their health (Fan et al., 2014), mostly due to the type and quantity of proteins and carbohydrates present (Lönnerdal, 2003). Indeed, various bioactive molecules in maternal colostrum and milk, including IgA, protect the newborn during the critical passage from the maternal environment to the outside world (Hasegawa et al., 2010; Kaetzel, 2014; Rogier et al., 2014a; Sato et al., 2014). Since the intestinal immune system develops slowly in offspring, a late transition occurs to the endogenous production of secretory (S) IgA, which is locally synthesized into the gut lumen. While the predominant protective mechanism of SIgA in breast milk likely involves the exclusion of pathogens and allergens at mucosal surfaces in the suckling infant by the immune system, other protective mechanisms could include the innate enhancement of the intestinal barrier and the promotion of a healthy commensal microbiota that limits the growth of potential pathogens (Rogier et al., 2014a). The long-term benefits on gut microbiota of early exposure to SIgA in breast milk were studied in a mouse model using a genomic (PhyloChip hybridization) approach (Rogier et al., 2014b). This study provided additional evidence of the benefits of breastfeeding, showing that SIgA in breast milk may promote lifelong intestinal homeostasis (Rogier et al., 2014b). In this investigation, we aimed to describe the multiplicity of the gut microbial community, and to monitor the influence of maternal milk nutrients (i.e., IgA) on the onset and shaping of the gut mucosal microbiota in the first weeks of life. In particular, we observed the development of the gut microbial community in pups born and breastfed by mothers, with or without IgA in their milk. We also focused on any possible accompanying effect of parental genetic background by means of cross-feeding experiments, where baby mice were breastfed by foster mothers with a different milk IgA profile with respect to their natural mothers.

Describing the composition of microbiota using a metaproteomic approach is a relatively new challenge that faces a number of problems not usually encountered in common shotgun proteomics analysis. The major issues in a metaproteomic study are the high degree of homology shown by closely related bacterial genomes, with the consequent large number of peptide sequences shared among several species, and the frequent absence of a proper protein database related to a specific host (Muth et al., 2013; Blackburn and Martens, 2016). In the last decade, only a few research groups have worked in this field, applying different data analysis pipelines, describing phylotype at a more or less specific taxonomic level, and using a public or *ad hoc* database, depending on the availability of metagenomics studies (Verberkmoes et al., 2009; Rooijers et al., 2011; Haange et al., 2012; Brooks et al., 2015). However, a number of bioinformatic tools and algorithms have been developed for the analysis of the large amount of information that have come out of high throughput liquid chromatography–tandem mass spectrometry (LC–MSMS) experiments (Mesuere et al., 2012, 2015; Tanca et al., 2014, 2015; Muth et al., 2015).

We recently reported a metaproteomics analysis of gut microbiota (GM) of newborn mice, where we used an original, semi-automated pipeline to produce a overview of the distribution of operational taxonomy units (OTUs), based on a count of the number of protein identifications (IDs) associated with bacterial taxa and microbiological filtering (Del Chierico et al., 2014).

In this study, a matrix assisted laser desorption ionization time-of-flight (MALDI–TOF) mass spectrometry (MS)-based culturomic analysis of mouse gut microbiota (MGM) was used, together with a bottom-up shotgun metaproteomics analysis, to compare resulting taxonomic charts, given the difference between the two approaches in terms of specificity and microbiome coverage. In a metaproteomic analysis, we improved the automation of our previously developed bioinformatic workflow, and continued to work on Mascot search output and to provide an alternative representation for the Lowest Common Ancestor (LCA) algorithm (Qin et al., 2010).

## Materials and Methods

### Mouse Model Breeding: Parental Genetic Background and Offspring Generations for the Evaluation of Breastfeeding in Gut Microbiota Programming

In this study, 126 baby mice from: (i) Balb/c 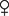 × Balb/c-Rag2^ko^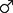 (hereafter named BALB; Spanopoulou, 1996), 45 individuals; (ii) Rag2^ko^ 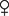 × Balb/c 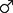, 45 individuals called RAG; (iii) Balb/c babies fed by Rag2^ko^ mothers, 18 individuals; and (iv) Rag2^ko^ babies fed by Balb/c mothers, 18 individuals; were maintained in conventional pathogen-free conditions and nursed either by their own or wet-nursing mothers (i.e., a cross-feeding experimental setting). They were then sacrificed and whole intestines recovered and processed prior to microbiological procedures for the characterization of gut mucosal and fecal microbiota. The MS-linked microbiological operational algorithm included: (a) MALDI–TOF MS-based culturomic analyses on mice sacrificed on days 3, 7, 14, and 21 for (i) and (ii) offspring, and days 3 and 7 for (iii) and (iv) offspring (nine individuals from both a Balb/c and Rag2^ko^ maternal genetic background); (b) metaproteomic nano-HPLC/MSMS analysis on days 3, 7, and 14 for (i), and (ii) (18 individuals, three biological replicates for every time point), and days 3 and 7 for (iii) and (iv) (12 individuals, three biological replicates for every time point); and (c) mouse gut database generation and a semi-automatic search engine for phylotype generation. In order to assess the distribution of circulating microbes in the mouse community, stools from 10 adult mice (five Balb/c and five Rag2^ko^ mothers) were processed by MALDI–TOF MS-based culturomic analysis.

All procedures with animals were performed in compliance with the relevant laws and local institutional guidelines at the Centro Ricerche Sperimentali, Istituto Regina Elena (Rome, Italy).

### MALDI–TOF MS-Based Culturomic Procedures

Bacterial recovery from whole intestines, tissues and fecal contents was performed as described by Del Chierico et al. (2014). Briefly, intestines were mechanically homogenized, washed and resuspended in Hanks’ balanced salt solution (HBSS) to obtain the enriched bacterial suspensions (EBSs). Ten microliters of 500 μL of an EBS were cultured for 48–72 h under aerobic, microaerophilic, and anaerobe growth conditions. Bacterial cell density was estimated by serial dilutions and plate counting. Based on morphology and growth conditions, colonies were characterized and re-isolated in order to proceed with MALDI–TOF MS-based IDs performed with a Microflex LT mass spectrometer (Bruker Daltonics, GmbH, Bremen, Germany) using Flex Control (version 3.0) and MALDI Biotyper automation control (version 2.0) software. Specifically, bacterial cells were directly picked from isolated colonies, smeared in triplicate onto an MSP 96 polished steel target (Bruker Daltonics) and overlaid with α-cyano-4-hydroxycinnamic acid matrix (Bruker Daltonics). Spectral analyses and bacterial IDs were automatically performed by matching against a reference library (version 2.0 SR 1; Bruker Daltonics; Del Chierico et al., 2014).

### Metaproteomic Procedures

A monodimensional nano-HPLC approach was applied to peptide separation before data-dependent acquisition (DDA) on a Triple TOF analyzer. Two hundred microliters of 500 μL of an EBS were added to 800 μL of sample buffer [7 M urea, 2 M thiourea, 40 mM Tris base, 4% CHAPS, 50 mM dithiothreitol (DTT)], pre-warmed at 37°C, sonicated for 20 s (5×) and then incubated for 1 h at 37°C. About 100 μg of protein were reduced with 50 mM DTT for 1 h at 37°C, followed by alkylation with 100 mM iodoacetamide (IAA) for 1 h at RT, and was finally digested overnight ON with 2 μL of 0.5 μg/μL of trypsin at 37°C. Three biological replicates of the tryptic digests were pooled and analyzed by nLC-MS on an Eksigent Ekspert NanoLC 400 system (Sciex, Toronto, ON, Canada) interfaced with a 5600+ TripleTOF (Sciex). Two μL (1 μg protein) of tryptic digests from each sample were injected in triplicate and pre-concentrated for 5 min on an Eksigent Trap column (350 μm × 0.5 mm Chrom XP C18, 3 μm, 120A nanoLC) at a flow rate of 5 μL/min. The gradient elution of peptides was performed on a C18-Acclaim PepMap 100 (25 cm, 75 μm I.D., 5 μm p.s., Thermo Fisher Scientific, Waltham, MA, USA), using a flow rate of 0.3 μL/min, at a temperature of 40°C, with eluents: (A) H_2_O/CH_3_CN 98:2 + 0.1% formic acid and (B) CH_3_CN/H_2_O 98:2 + 0.1% formic acid, and a gradient from 5 to 25% B in 120 min. MS data were acquired in information-dependent acquisition (IDA) mode. The 35 most intense precursor ions were selected for each survey scan, keeping the active exclusion enabled for 30 s after two MS^2^ scans on the same precursor. Raw data was processed by a Mascot Distiller, 2.5.1.0 version, performing a protein search against the NCBInr DB, with bacteria (eubacteria) as a taxonomic restriction (11,349,244 sequences) for microbial protein ID, and the SwissProt DB with *Mus musculus* as a taxonomic restriction (1,6724 sequences), to estimate the number of spectral features dedicated to mouse proteins. Error tolerances were set to 20 ppm and 0.01 Da for precursors and fragments, respectively. Cysteine carbamidomethylation was set as fixed modification and methionine oxidation as a variable modification. A single miscleavage was allowed. Results were filtered keeping the FDR below 1% with the Mascot Percolator, and were exported as csv files, including only hits with at least a bold red peptide, and the list of all proteins spanning the same peptide set.

**FIGURE 1:**
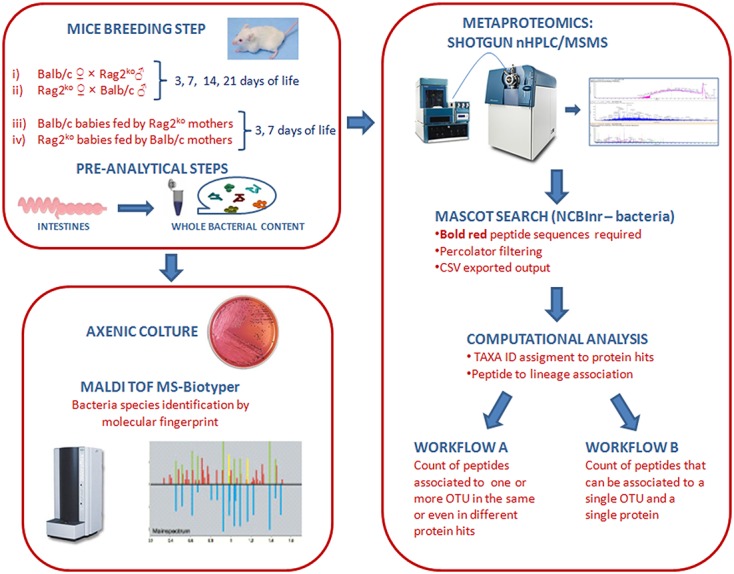
**Graphical representation of culturomics- and metaproteomics-based pipelines.** The scheme outlines the mouse breeding step, the pre-analytical phase and the two analytical methodologies, consisting of culturomics and metaproteomics pipelines.

Mouse gut microbiota phylotypes were grouped at different taxonomic levels by applying a modified version of the Excel macro developed by Del Chierico et al. (2014). More specifically, a script in Python Programming Language was created to implement the whole workflow of the original Excel macro, starting from the Mascot “csv” exported files to produce two different representations (i.e., **workflows A** and **B**) of the taxonomic distribution of the OTUs. **Workflow A** was obtained by counting the number of peptides associated with one or more taxa, namely, including peptides present in the same protein hit but associated with different OTUs, or peptides found in different protein hits and associated with the same OTU. **Workflow B** reported the number of peptides that could be associated with a single OTU only (**Figure 1**). To let the program work properly, a NCBI “taxon ID” was previously assigned to each NCBI “gi accession number” by a second Python script, then the whole taxonomy lineage was associated with each peptide entry, allowing the application of a counting workflow at every taxonomic level. A threshold value of 10 on the peptide score was set as an input for the script.

Once the OTUs list was obtained, manual filtering was performed to remove all OTUs that were found in a single technical replicate only. The number of peptides associated with each OTU were averaged among the three technical replicates, excluding those with a value lower than one. Taxa associated with a lower number of peptides were grouped in “other” categories to make histograms and pie charts clearer. The LCA algorithm was also applied to a selected number of samples using the Unipept web application^1^. The peptide list was uploaded, without duplicates and with the same threshold value of 10 on the peptide score as previously set, flagging the option “Equate I and L.”

The Shannon–Wiener index was calculated by the formula:

$$H^{\prime }\,=\;-\;\overset{R}{\underset{i=1}{\Sigma }}p_{i}\,ln\,p_{i}$$

where: R = richness; p*_i_* = number of individuals belonging to the *i*th generus in each sample.

Calculation and plotting was performed by Microsoft Excel software (Testa and Bojarski, 2009).

A Venn diagram of the OTUs shared by Balb/c, Rag2^ko^, Balb/c fed by Rag2^ko^, and Rag2^ko^ fed by Balb/c mice was generated by the web application, Venny 2.1^2^.

Mass spectrometry proteomics data was deposited with the ProteomeXchange Consortium via the PRIDE (Vizcaíno et al., 2016) partner repository, using a dataset identifier PXD004033.

## Results

### Mouse Model Breeding: Parental and First Filial Generations

In our experimental pipeline we chose to investigate the MGM in pre-weaned mice in relation to age, parental genetic background and the presence of IgA in maternal milk. For this purpose, we used Balb/c wild-type (WT) and Rag2^ko^ mice. The latter, due to the deletion of the *RAG2* gene, were unable to recombine immunoglobulin and T cell receptor DNA fragments and for this reason lacked T and B cells and all immunoglobulins, including IgA in breast milk (Spanopoulou, 1996). Therefore, in the mouse breeding panel, the parental (P) generation was represented by immunocompetent Balb/c and immune-deficient Rag2^ko^ mice, while the F1 generation provided immunocompetent offspring. First filial (F1) mice were fed by their own mother (either Balb/c or Rag2^ko^). The cross-feeding F1 generation was obtained by exchanging mothers at day 1 of life. Thus, pups born to Balb/c mothers were fed by Rag2^ko^ foster mothers and vice versa.

### Culture-Based MALDI-TOF MS Approach to Assess MGM Microbial Species Distribution

The characterization of the MGM of adult and baby mice by MALDI–TOF MS Biotyper allowed an assessment of bacterial IDs at both the phylum and species levels. In particular, the gut microbial composition of WT and Rag2^ko^ P mothers, and that of F1 newborns was investigated, the latter at 3, 7, 14, and 21 days of life, while their respective cross-fed offspring (pups born to Balb/c and Rag2^ko^ mothers fed by Rag2^ko^ and Balb/c foster mothers, respectively) were studied at 3 and 7 days of life only. In a previously standardized procedure (Del Chierico et al., 2014), and after a 48–72 h growth period for every subset of mice and at each time point after birth, we selected and picked an average of 30–40 distinct individual microbial colonies, each characterized by different morphological (e.g., size, shape, and color characteristics) and metabolic features (e.g., aerobic, anaerobic, microaerophilic conditions, and differential growth factor requirements).

**FIGURE 2:**
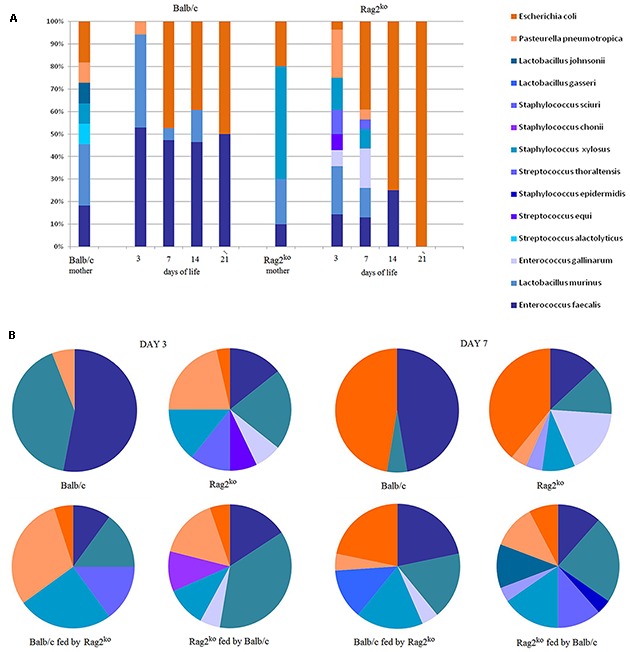
**Axenic culture-based analysis of mouse gut microbiota at the species level. (A)** A histogram representing the relative abundance of mouse gut microbiota (MGM) species from mothers’ fecal contents, and Balb/c and Rag2^ko^ baby mice intestinal contents at 3, 7, 14, and 21 days. **(B)** Pie charts showing the distribution of species in the MGM of offspring at days 3, and 7. Phyla are identified by color shades, specifically blue for *Firmicutes* and orange for *Proteobacteria*.

The MGM of WT and Rag2^ko^ mothers displayed similar levels of *Firmicutes* and *Proteobacteria*, with a relative distribution of approximately 75 and 25%, respectively. The composition of the gut microbiota of baby mice from Balb/c and Rag2^ko^ mothers appeared quite similar to that of their respective parents after 3 days of life, even if the predominance of *Firmicutes* over *Proteobacteria* was more marked in Balb/c than in Rag2^ko^ mice (95 and 75%, respectively). At the following time points (from 7 to 21 days) in babies of Balb/c mothers, MGM composition rapidly stabilized with approximately 50% *Firmicutes* and 50% *Proteobacteria*. However, in pups born to Rag2^ko^ females, it gradually changed with a progressive disappearance of *Firmicutes* and an increase in *Proteobacteria* levels eventually accounting for 100% of the MGM (**Figure 2A**).

Significant differences were also observed at the species level. In Balb/c mothers, the presence of lactic acid bacteria (LAB), represented by *Enterococcus faecalis*, *Lactobacillus murinus*, and *Lactobacillus johnsonii* (overall 55%), was higher than in Rag2^ko^ mothers (%). In contrast, *Escherichia coli* (about 20%) was found less frequently. In Balb/c mothers, *Streptococcus alactolyticus* accounted for 10% of the identified species. Remarkably, a different MGM composition in Balb/c WT and Rag2^ko^ mothers was also detected with regard to the level of opportunistic bacterial pathogens (OBPs), such as *Pasteurella pneumotropica* and *Staphylococcus xylosus*. In particular, we observed a very low OBP level (less than 20%) in Balb/c mothers, and almost the opposite pattern (up to 50% of OBP, entirely associated with *S. xylosus*) in Rag2^ko^ mothers (**Figure 2A**).

From days 3 to 21 of life, the MGM of baby mice born to a Balb/c mother included mostly LAB, mainly represented by *E. faecalis* and *L. murinus*, ranging from 90% of the overall identified bacterial isolates at 3 days of life, to 50% at 21 days of life (**Figure 2A**). In particular, we found a progressive replacement of *L. murinus* (30% at 3 days) with *E. coli* (50% since day 14). Overall, after 7 days of life, the MGM of the babies of a Balb/c mother contained mostly LAB, similar to the MGM of adult Balb/c mice. On the other hand, the population of LAB in pups of a Rag2^ko^ mother was reduced, ranging from 25% at 3 days (mainly ascribed to *Enterococcus gallinarum* and *L. murinus* isolates) to 0% at 21 days, while at the same time points *E. coli* increased from less than 5–100% of the overall identified bacteria. A high level of OBP bacteria (approximately 45%), due to the presence of *P. pneumotropica*, *Streptococcus equi* and *S. xylosus* bacterial species, was observed at 3 days in mice born to Rag2^ko^ females.

To complete our observations on the effect of modulating feeding on MGM rebiosis, a maternal cross-feeding scheme was used to assess the possible occurrence of LAB restoration or OBP maintenance in F1 offspring. Therefore, we analyzed the offspring of Rag2^ko^ or Balb/c baby mice fed by Balb/c and Rag2^ko^ foster mothers, respectively (cross-feeding). Since, during pregnancy, immunocompetent mothers transfer their serum IgG to pups through the placenta, we designed cross-feeding experiments to separate the impact of serum IgG and milk IgA on neonatal MGM composition. At day 3, Rag2^ko^ mice fed by Balb/c mothers showed an MGM similar to that of Rag2^ko^ mice fed by their own mothers. The presence of *E. faecalis* and *L. murinus* was predominant but not as much as in Balb/c mice, while *Proteobacteria*, mainly represented by *P. pneumotropica* and *E. coli*, was around 25%. At day 7, their MGM composition began to differ from that of Rag2^ko^ mice, showing a lower abundance of *E. coli* and a higher presence of *Firmicutes*. Specifically, we observed: (i) an increase in *S. xylosus*, (ii) the appearance of *Staphylococcus sciuri, Streptococcus thoraltensis*, and (iii) a reduction in *L. animalis* and *E. faecalis*. These results suggest that IgA supplementation promotes the exclusion of some OBP, such as *P. pneumotropica*, and the temporary appearance of beneficial *S. thoraltensis* species in pups born from Rag2^ko^ mothers (**Figure 2B**).

At day 3, Balb/c baby mice fed by Rag2^ko^ mothers showed an MGM composed of a high percentage of *P. pneumotropica* and *S. xylosus*, and a less abundant population of *L. murinus*, *S. thoraltensis*, *E. coli*, and *E. faecalis*. Four days later, the proportion of *Firmicutes* was found to be higher in cross-fed mice than in naturally fed mice; the MGM was populated by *S. sciuri*, *S. xylosus*, *E. gallinarum*, *P. pneumotropica*, *L. murinus*, *E. faecalis*, and *E. coli* (**Figure 2B**). All described species belonged to *Proteobacteria* and *Firmicutes*. Species from *Verrucomicrobia*, *Synergistetes*, *Spirochaetes*, *Bacteroidetes*, *Actinobacteria*, and *Fusobacteria* were not isolated by a MALDI-TOF MS biotyper culture-based approach, probably because of the requirement for restrictive, *in vitro* culture conditions. However, cultivable fractions of bacteria were consistent with those described in pivotal papers on MGM onset and development (Schaedler et al., 1965; Lee et al., 1971).

### Taxonomic Description of MGM by a Metaproteomics Approach

To support and extend the results we obtained with MALDI–TOF MS Biotyper analysis, we applied a shotgun metaproteomics approach on a sub-set of samples, trying to describe the MGM composition in terms of OTUs, and focusing on Balb/c and Rag2^ko^ baby mice at 3, 7, and 14 days of life, and respective cross-fed mice at 3 and 7 days. Three biological replicates were pooled together for all 10 conditions and analyzed by nano-HPLC/MSMS with monodimensional chromatographic runs and DDA acquisitions on a nanoESI–Triple TOF mass spectrometer.

We observed a fair variability in the number of identified bacterial proteins among the samples, ranging from a maximum of 931 filtered hits (3397 peptide sequences) for a Rag2^ko^ sample at day 7, to a minimum of 150 (329 peptide sequences) for a cross-fed sample at day 3 (**Supplementary Table S1**). Instead, general good reproducibility was observed among the three technical replicates, except for a couple of runs (**Supplementary Table S2**).

**FIGURE 3:**
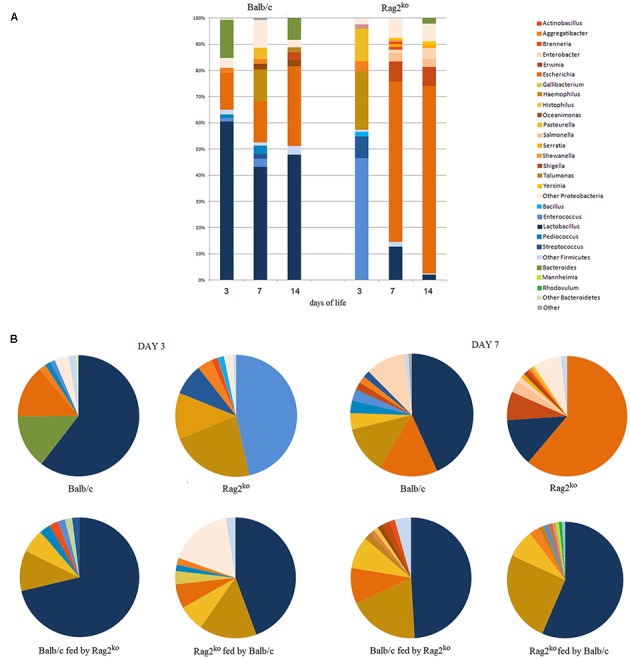
**Metaproteomics analysis at the genus level by workflow B. (A)** A histogram representing MGM relative abundance at the genus level, from Balb/c and Rag2^ko^ baby mice biopsy contents at days 3, 7, and 14. **(B)** Pie charts comparing the MGM content in Rag2^ko^ and Balb/c mice with that of their respective, cross-fed offspring at days 3 and 7. Phyla are identified by color shades, specifically blue for *Firmicutes* and orange for *Proteobacteria*.

We obtained taxonomic charts, employing two different approaches (**workflows A** and **B**) for each taxonomic level (**Supplementary Table S3**). The two representations showed the same distribution of taxa at a generic taxonomic level (e.g., phylum, order), but when considering more specific levels in the lineage (e.g., family, genus, and species), *Firmicutes* were almost exclusively represented by the *Lactobacillus* genus, while there was a large number of different genera belonging to *Proteobacteria*, sharing a considerable portion of the genome (e.g., *Escherichia/Shigella*). In the present work, the outcomes of the two counting procedures showed a comparable trend in the evolution of the MGM in mice pups, and we thus chose to use the more restrictive one (**workflow B**), which was also more similar in concept to the LCA approach (**Figure 3**; **Supplementary Figure S1**).

The histogram in **Figure 3A** shows how the distribution of OTUs was very similar to that obtained by a culturomics approach, shown in **Figure 2A**. The composition of the MGM seemed to be stable in the first 2 weeks of life for pups of Balb/c mothers. *Firmicutes* slightly decreased at 3 and 7 days, but then the ratio *Firmicutes*/*Proteobacteria* remained constant over time. *Escherichia* was instead the dominant genus in the babies of Rag2^ko^ mothers after the first week of life.

With regard to the microbiota profile composition in cross-fed offspring at days 3 and 7, we found that in the pups of Balb/c females fed by Rag2^ko^ foster mothers, the microbiota profile composition looked similar to that of Balb/c mice, considering *Firmicutes* proportion, rather than to Rag2^ko^ mice, with a prevalence of LAB (**Figure 3B**). At day 7, the evolution in the general profile was similar to that of Balb/c mice fed by their own mothers. Lactobacilli were also present in the MGM of pups born to Rag2^ko^ mothers fed by Balb/c mothers, and the *Firmicutes*/*Proteobacteria* ratio was similar to that of neonates born to Rag2^ko^ females at 3 days of life. At day 7, an increment in the population of *Lactobacillus* was observed together with a decrease of *Escherichia*, which, on the contrary, was the predominant genus in pups of Rag2^ko^ mothers fed by their own mothers.

**FIGURE 4:**
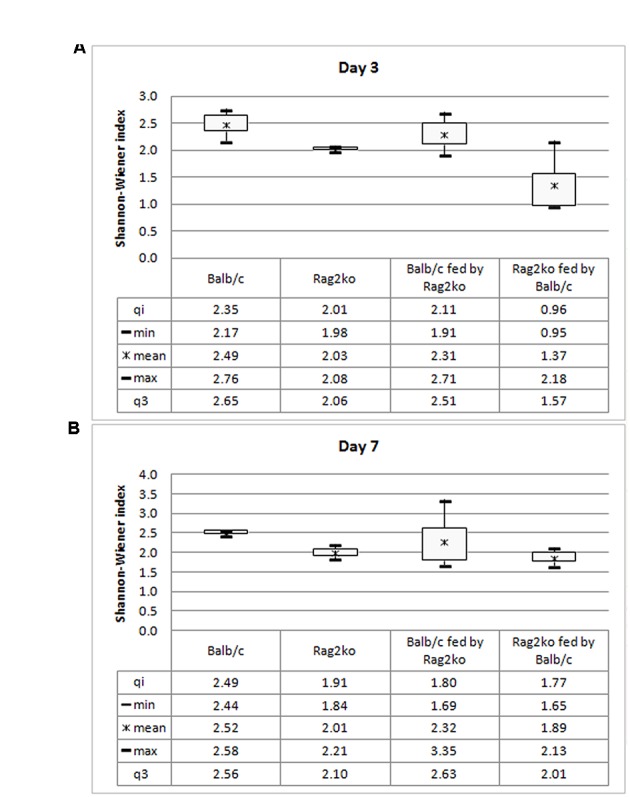
**Diversity index.** Box plot of mean values of the Shannon–Wiener index for Balb/c, Rag2^ko^, Balb/c fed by Rag2^ko^, and Rag2^ko^ fed by Balb/c mice groups at a genus level at days 3 **(A)** and 7 **(B)**.

The Shannon–Wiener diversity index is a quantitative measure of the alpha diversity in an ecological community such as microbiota. In this study, we used this index to take into account how the microbiota genera were distributed among Balb/c, Rag2^ko^, Balb/c fed by Rag2^ko^, and Rag2^ko^ fed by Balb/c mice at 3 and 7 days (**Figure 4**) of mouse life. At day 3, the value of the diversity index increased in both Balb/c mice and Balb/c mice fed by Rag2^ko^ mice, reflecting an increase of ecology evenness. In Rag2^ko^ mice, and more so in Rag2^ko^ mice fed by Balb/c mice, the indices were low, indicating less diversity in terms of OTUs in these groups (**Figure 4A**). At 7 days, in Rag2^ko^ mice fed by Balb/c mice, the index was increased, while in the remaining three groups it remained fairly stable (**Figure 4B**).

## Discussion

### Axenic Culture-Based MALDI–TOF MS Study

The MGM of pre-weaning mice is relatively simple and uniform (Hasegawa et al., 2010; Del Chierico et al., 2014; Hasegawa and Inohara, 2014). However, OTUs of very early gut microbiota correlated with age and parental genetic backgrounds during early life stages (Koren et al., 2012; Del Chierico et al., 2014; Carmody et al., 2015). The GM of WT, Balb/c, and Rag2^ko^ mice exhibit different microbial compositions due to a drastic difference in their respective immune systems (Shulzhenko et al., 2011). In fact, Rag2^ko^ mice are severely immunodeficient because the *RAG2* (Recombination activating gene 2) mutation blocks the rearrangement of immunoglobulin and T-cell receptor genes, thus impairing the development of B and T cells and the production of all antibody isotypes, including IgA (Spanopoulou, 1996). IgA serves as a first line of defense in the protection of the intestinal epithelium from enteric toxins and pathogenic microorganisms; it influences the composition of gut microbiota, promotes the translocation of antigens across the intestinal epithelium to the gut-associated lymphoid tissue (GALT), and down-regulates pro-inflammatory responses (Hasegawa et al., 2010; Liu and Rhoads, 2013). At birth, newborn mice are still unable to produce IgA and they rely on passively acquired IgA contained in breast milk to shape the composition of their developing gut microbiota and protect themselves against infections (Tsuji et al., 2008; Mantis et al., 2011; Shulzhenko et al., 2011; Rogier et al., 2014a,b). The presence of LAB is higher in the stool content of Balb/c than in Rag2^ko^ mothers (Hemme et al., 1980; Inoue et al., 2007; Kaburagi et al., 2007). LAB form a substantial part of the gut microbiota of rodents in their first days of life and stay on as a dominant microbiota component throughout the whole life of the animal. Indeed, LAB are believed to play an important role in the host’s well-being (Vaughan et al., 2002). In Balb/c mothers, the obvious presence of *S. alactolyticus*, a bacterial species involved in the suppression of intestinal pathogens’ growth and in the enhancement of the immune functions of mice, was noted (Hudault et al., 1997; Vitiñi et al., 2000; Won et al., 2002). Remarkably, we found a lower level of OBP in the MGM composition of Balb/c WT mice compared to Rag2^ko^ mothers (Literák and Mráz, 1991; Won et al., 2002; Fox, 2007).

The differential distribution of LAB and OBP, especially evident in Balb/c WT mothers (high LAB/low OBP) and Rag2^ko^ mothers (low LAB/high OBP), has led to the hypothesis that IgA modulates the differential propagation and/or suppression of OBP and LAB in adult mice, favoring OBP colonization. Several commensal groups show identical species that colonize the human and mouse digestive tracts (e.g., *Enterobacteriaceae*), however the MGM has many unique features, namely: (i) a low abundance of oral obligate anaerobes, (ii) a high abundance of ileal segmented filamentous bacteria that induce a specific immune response, (iii) a low abundance of Bifidobacteria which affect the host’s susceptibility to infection by pathogens, and (iv), a different abundance of *Lachnospiraceae* species (Hasegawa et al., 2010; Hasegawa and Inohara, 2014).

In our model, specifically in F1 MGM analysis, the MGM of Balb/c and Rag2^ko^ baby mice showed a very similar composition to their respective mothers after 3 days of life, with a predominance of *Firmicutes* and low levels of *Proteobacteria*. This is not surprising as it is known that newborn mice acquire their microbiota from their mothers during delivery (Hansen et al., 2012). At subsequent time points, *Firmicutes* and *Proteobacteria* reached a stable level of 50% in Balb/c baby mice, while in Rag2^ko^ pups, *Proteobacteria* levels progressively increased, reaching 100% at 21 days.

At the species level, we noticed a uniform MGM LAB profile, probably related to the 3–21 days’ breastfeeding period. Indeed, the observed transition in the MGM just before weaning (i.e., day 21), when mice start to also eat conventional mouse food, confirms the important relationship between the feeding type and the composition of the commensal community as LAB are lactose-fermenting facultative anaerobic organisms linked to breastfeeding (Hasegawa et al., 2010).

The presence of a high level of OBP in both Rag2^ko^ adults and baby mice may be the result of the absence of IgA. Thus, it may be hypothesized that IgA has a crucial role in regulating the composition of the MGM, maintaining a correct balance between LAB and OBP and controlling the proliferation of *E. coli*.

In Rag2^ko^ baby mice fed by Balb/c mothers at day 3, the phyla portions are similar to Rag2^ko^ mice, while at day 7 *Firmicutes* were higher compared to normally fed baby mice. With regard to Balb/c mice fed by Rag2^ko^ mothers, at day 3 the MGM of these mice resembles more to that of Rag2^ko^ mice, considering the *Firmicutes*/*Proteobacteria* ratio, while at day 7, the relative abundance of *E. coli* increased but still resulted in a lower level than that of normally fed mice. Most probably, IgA supplementation due to nursing by Balb/c mothers promotes the exclusion of some OBP and the temporary appearance of beneficial species (Carlisle et al., 2011).

### Metaproteomic Analysis

The metaproteomic study was centered on a restricted set of samples with respect to a culture-based approach. For the phylotype description of the MGM, we used a modified version of the semi-automated pipeline previously employed in other investigations, focusing our analysis on the peptide count level and working on the Mascot output after searching against the NCBI nr database (Del Chierico et al., 2014).

Databases that consider the host environment in which the microbial community is established are recommended, when feasible, for protein search and ID in order to reduce as much as possible the number of false OTU IDs that can come out with highly annotated public repositories. Moreover, such a search requires deep revision and microbiological filtering, and often leaves uncertainty in the actual reliability of the OTU IDs with respect to the district under investigation and the host, due to the high degree of gene homology expressed in the whole bacterial (eubacterial) kingdom, and the consequent high number of orthologs. This is also the reason why it is hard to produce a reasonable taxonomic chart going down through the lineage at the species or even the genus level, as the number of specifically identifying peptides could be too low. We tested the customized DB used in a study on a murine fecal metaproteome (Tanca et al., 2014), obtaining results on bacterial sequences almost overlapping with the NCBInr DB, so we decided not to use it (data not shown). Moreover, this DB was not suitable for our computational pipeline as it does not report the NCBI accession number for proteins, which we use to assign the lineage to peptides, and it produces an output without all the orthologs related to each identified metaprotein, which is crucial information for our counting workflows.

With regard to the metaproteomic phylotype description, the LCA approach is now the most widely used, providing a overview of microbiota composition based on peptide sequences that can be unambiguously associated to an OTU at a given taxonomic level (Tanca et al., 2014, 2015). The easily accessible web application, Unipept (Mesuere et al., 2012), yields a graphic representation of OTU distribution at each taxonomic level by simply submitting a peptide list. The metagenomic software, MEGAN (Rudney et al., 2010), can be used for metaproteomic data as well. Even though MEGAN is a more time-consuming procedure and requires a bigger computational effort, it provides a phylotype description of the microbiota based on the LCA, and also allows easy access to the EggNOGG or SEED pathway databases for a functional analysis (Rudney et al., 2010). The recently developed metaproteome analyzer (MPA) is an open-source software that allows a complete analysis of metaproteomic data, and performs database searches, taxonomic descriptions, protein groupings and assignments to pathways (Muth et al., 2015). This software relies on the robust LCA algorithm to describe the taxonomic distribution of OTUs. The analysis can be performed with a series of rules that can be applied separately or together, yielding different results. Unfortunately, the portable version of the software is currently unable to manage large data-sets such as ours, or to work with huge DBs without computational drawbacks.

Our two different workflows (**A** and **B**), aimed at facing the problem of the extended homology of the microbial community, made it difficult to produce taxonomic charts. Mascot searches on a huge DB, such as NCBI nr bacteria, yielded a large number of protein hits. However, a high percentage of proteins was associated with the same set or just sub-set of peptides, which were found in other proteins with a higher ranking position. Excluding all the hits that do not have at least a specific top-ranking peptide (at least a bold-red peptide in the Mascot protein hit) that can justify a match with an OTU, we risked under-evaluating the presence of some taxa but, on the other hand, we eliminated a large number of false IDs. The outcomes from **workflows A** and **B** were very similar at the phylum level, but, when considering family or genus, the results became divergent. Apart from the different number of peptides that were included in the counts, the main reason for this divergence was the degree of homology in a particular taxonomic branch. Thus, a strict representation, such as one considering only unique and taxon-specific peptides to determine the presence of an OTU (**workflow B**), could be misleading under a “quantitative” point of view, but be more reliable under a “qualitative” point of view. However, by counting all peptides matching one or more OTUs (**workflow A**), we surely over-estimated the presence of some taxa, including also genera that did not actually exist in the mouse gut, but we provided an overall picture, which is more indicative of the abundance of OTUs in the MGM. Therefore, to properly analyze the outcomes of our bioinformatic pipeline at a specific taxonomy level, both representations should be taken into consideration to have a complete idea of the distribution of OTUs based on peptide ID. Moreover, comparing the analysis of peptides obtained by our workflow versus that resulting from the LCA algorithm, we found either identical, or even completely divergent results. The reason can be ascribed to different filtering criteria and to the total number of families associated with the taxonomic branch. In particular, the larger the number of genera belonging to the same branch, the higher the number of shared peptides among these genera. For example, *Escherichia*, which belongs to the wide *Enterobacteriaceae* family, is often under-represented with the LCA approach as it shares many homolog peptides with other genera of the same family (i.e., *Shigella*, *Yersinia*, *Salmonella* etc.), and the number of taxon-specific peptides is generally low (**Supplementary Figure S2**).

OTUs distribution in Rag2^ko^ and Balb/c baby mice in the first 2 weeks of life resembled those obtained with the culturomic approach, with a progressive increase of *Proteobacteria*, mostly represented by *Escherichia*, for the former and the reaching of a stable *Firmicutes*/*Proteobacteria* ratio after 3 days of life. The analysis of cross-fed offspring showed that at days 3 and 7, the microbiota profile composition of pups from Balb/c mothers fed by Rag2^ko^ foster mothers looked similar to that of Balb/c mice, suggesting the restoration of MGM symbiosis conditions (Mao and Franke, 2015). Furthermore, in the MGM of Rag2^ko^ mice fed by Balb/c mothers, the *Firmicutes*/*Proteobacteria* ratio seemed to be consistent with that of Rag2^ko^ mice at 3 days of life. At day 7, we observed an increment in the population of *Lactobacillus*, together with a decrease of *Escherichia*, which was the predominant genus in Rag2^ko^ mice fed by their own mothers.

**FIGURE 5:**
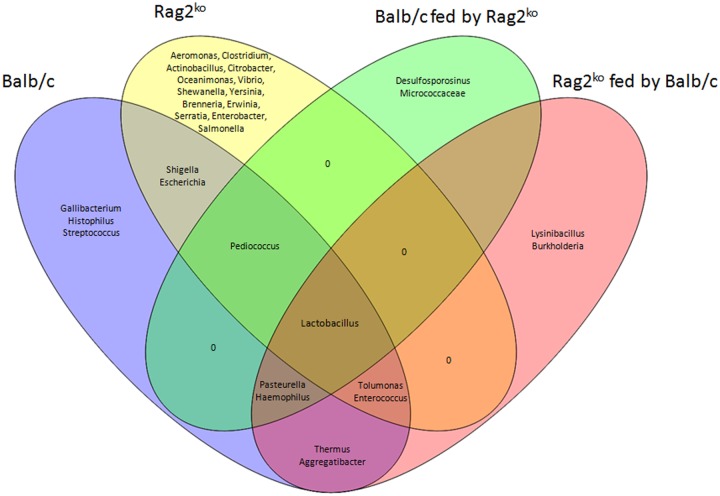
**Operational taxonomy unit correlation.** Venn diagram showing the operational taxonomy units (OTUs) shared by Balb/c, Rag2^ko^, Balb/c fed by Rag2^ko^, Rag2^ko^ fed by Balb/c mice groups.

We finally attempted the functional classification of OTUs and their comparison in groups by means of a Venn diagram that would provide us an outline of the possible relationship between OTUs and different sample sets (**Figure 5**). The diagram showed that the only OTU shared by all groups was *Lactobacillus*, while *Pediococcus* was shared amongst Balb/c, Rag2^ko^ and Rag2^ko^ fed by Balb/c. *Aeromonas*, *Clostridium*, *Actinobacillus*, *Citrobacter*, *Oceanimonas*, *Vibrio*, *Shewanella*, *Yersinia*, *Brenneria*, *Erwinia*, *Serratia*, *Enterobacter*, and *Salmonella* were present only in Rag2^ko^ mice, while *Gallibacterium*, *Histophilus*, and *Streptococcus* were exclusively in Balb/c mice. The two groups shared *Shigella* and *Escherichia*.

Analyzing cross-fed individuals, we found that *Desulfosporosinus* and *Micrococcaceae* belonged to Balb/c mice fed by Rag2^ko^ mice, while *Lysinibacillus* and *Burkholderia* belonged to Rag2^ko^ mice fed by Balb/c mice, and that the two groups did not share any OTUs. Moreover, *Pasteurella* and *Haemophilus* were common between Balb/c mice and Balb/c mice fed by Rag2^ko^ mice, while *Tolumonas* and *Enterococcus* were common between Rag2^ko^ mice and Rag2^ko^ mice fed by Balb/c mice, highlighting a relationship between these OTUs and the genetic background of the mice.

The two MS-based approaches we employed in this work provided us with two different points of view in the analysis of MGM composition. We observed good agreement between the two approaches in the description of the evolution of the microbial community of Balb/c and Rag2^ko^ mice pups in the first 2 weeks of life (**Figures 2A** and **3A**). In the case of cross-feeding experiments, we observed more variance; however, differences between the two techniques in terms of taxonomic specificity and proteome coverage must, regardless, be taken into account. The use of one method does not preclude the application of the other one, with a sort of complementarity that provides a thorough description of OTU distribution in a microbiota.

## Conclusion

Our study showed that after 3 days of life, the microbiota composition of Balb/c and Rag2^ko^ baby mice differed from each other, but resembled the respective microbiota of the mothers. Such a difference was particularly evident when analyzing the GM in terms of LAB and OBP distributions: in the presence of IgA in milk, we found a high proportion of LAB and a low proportion of OBP (Balb/c), while in milk where IgA was absent, we found the opposite distribution (Rag2^ko^). In subsequent days, differences between the two kinds of mice became more pronounced, supporting the hypothesis of an influence of the milk composition in shaping microbiota. However, after cross-feeding experiments we did not observe a complete restoration of the Balb/c or Rag2^ko^ microbiota phenotype, observing similar evolutions in both cases. This can probably be ascribed to the combined effect of both breastfeeding and a sort of microbiota inheritance achieved by newborns during parturition. It is known that during delivery, babies can acquire their mother’s microbiota. This represents a pre-adapted and “ready-to-use” microbiota inoculum, constituting an already mature and stable niche that preempts the space, and excludes or inhibits new pioneers (Del Chierico et al., 2015). Our hypothesis of a functional link between the presence/absence of IgA and modulation of the gut MGM should now be reinforced by further investigations on newborn immunological programming, with the aim of implementing the interplay between the early programming of MGM and Ig generation in terms of the “microbiota organ.”

We also confirmed the value of our bioinformatic pipeline, finding consistency between our results and those of LCA analysis when they are made at a less specific taxonomic level, such as phylum or order. The differences observed at more specific taxonomic levels suggest a further point of view for data interpretation. Functional and quantitative analyses will be the next, fundamental steps to take to complete our metaproteomic workflow for future investigations.

## Author Contributions

SM performed all the LC–MS experiments, data processing and the metaproteomic analysis. He also worked at the development of the bioinformatic pipeline and paper writing. FC attended to bacterial culture and performed all MALDI-TOF Biotyper spectra acquisitions. She was also involved in the bioinformatic pipeline development and had an important role in the microbiological interpretation of results. She wrote the corresponding sections of the manuscript. PV was involved in the sample preparation, from cell lysis to protein digestion. She also had a role in the metaproteomic analysis of LC–MS data. MR was responsible of the mice pups selection, sacrifice and intestine tract extraction. AC had a crucial role in the automation of the bioinformatic pipeline and its development. MC edited a part of the Python scripts for the improvement of the automated bioinformatic workflow. LuP helped in sample preparation. AU was involved in the experimental design and metaproteomic analysis. RC was involved in the experimental design and results interpretation. IL was involved in the manuscript writing and editing. BD was involved in the manuscript writing and editing. LoP led the experimental design. She was involved in the metaproteomic analysis and microbiological data interpretation. She supervised the manuscript writing and editing.

## Conflict of Interest Statement

The authors declare that the research was conducted in the absence of any commercial or financial relationships that could be construed as a potential conflict of interest.

## Footnotes

1 http://unipept.ugent.be/

2 http://bioinfogp.cnb.csic.es/tools/venny/index.html

## Supplementary Material

FIGURE S1 **Metaproteomics analysis at genus level by workflow A. (A)** The histogram represents mouse gut microbiota (MGM) relative abundance at the genus level, from Balb/c and Rag2^ko^ baby mice biopsy contents at days 3, 7, and 14. **(B)** Pie charts comparing the MGM content in Rag2^ko^ and Balb/c mice with that of their respective cross-fed offspring at days 3 and 7. Phyla are identified by color shades, specifically blue for *Firmicutes* and orange for *Proteobacteria*.

FIGURE S2 **Comparison between workflow B and Lowest Common Ancestor analysis.** When representing operational taxonomy unit (OTU) distribution at the genus level, the picture that came from the **workflow B** counting procedure and the Lowest Common Ancestor (LCA) algorithm was very similar or even remarkably different, due to the degree of ramification of the respective family. The *a priori* filtering of our workflow increased the number of taxon-specific peptides for genera sharing a large portion of the genome with others.

TABLE S1 **Mascot search results for all samples**.

TABLE S2 **Summary of spectral analysis and FDR values**.

TABLE S3 **Counting workflow output**.
