# Supplementary figures and images for: Monitoring Perinatal Gut Microbiota in Mouse Models by Mass Spectrometry Approaches: Parental Genetic Background and Breastfeeding Effects
Source: Front Microbiol. 2016 Sep 26;7:1523. doi: 10.3389/fmicb.2016.01523 (PMC5036385; doi:10.3389/fmicb.2016.01523)

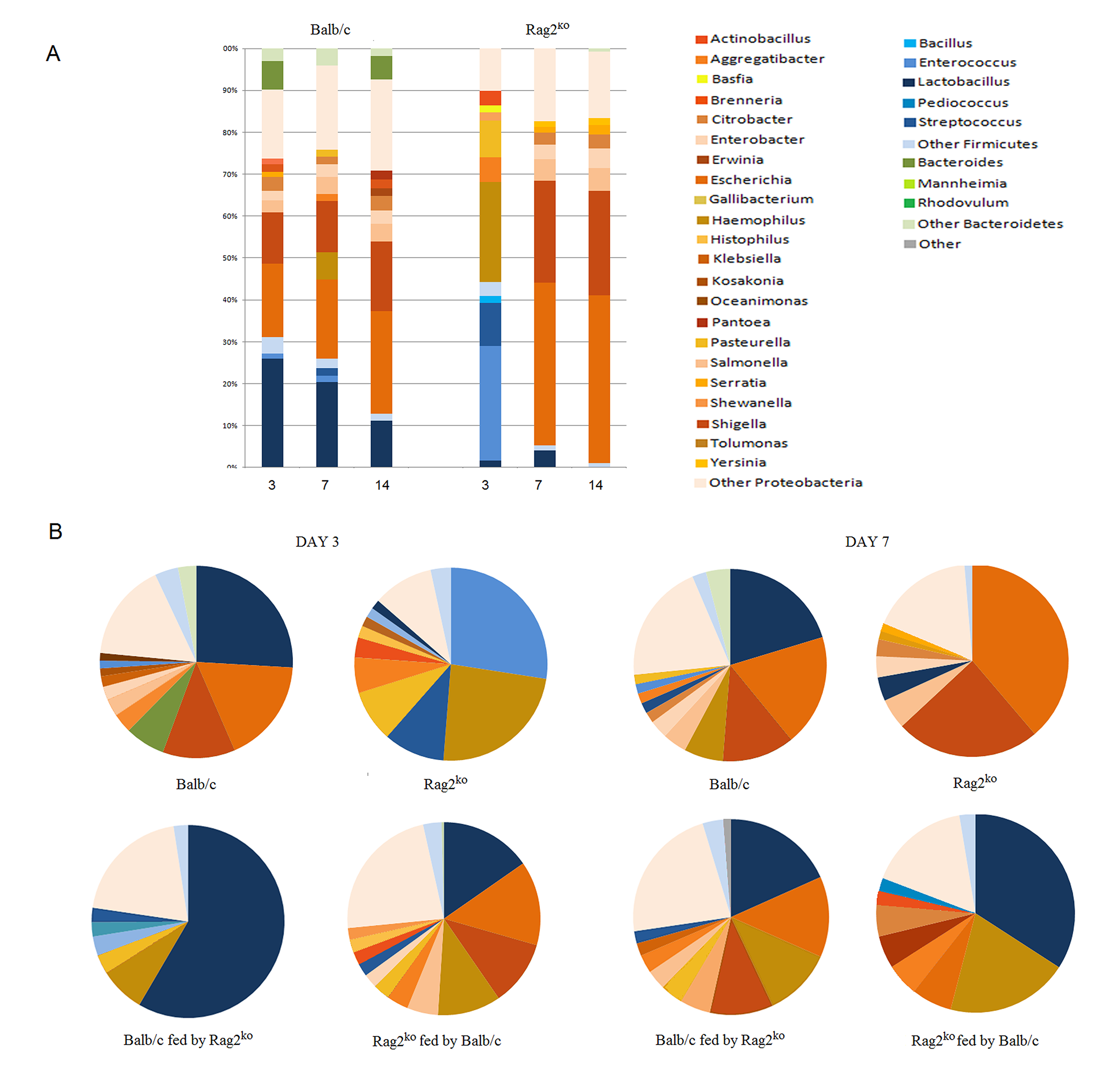

Supplement: FIGURE S1 — Metaproteomics analysis at genus level by workflow A. (A) The histogram represents mouse gut microbiota (MGM) relative abundance at the genus level, from Balb/c and Rag2ko baby mice biopsy contents at days 3, 7, and 14. (B) Pie charts comparing the MGM content in Rag2ko and Balb/c mice with that of their respective cross-fed offspring at days 3 and 7. Phyla are identified by color shades, specifically blue for Firmicutes and orange for Proteobacteria. [file Image_1.TIF]

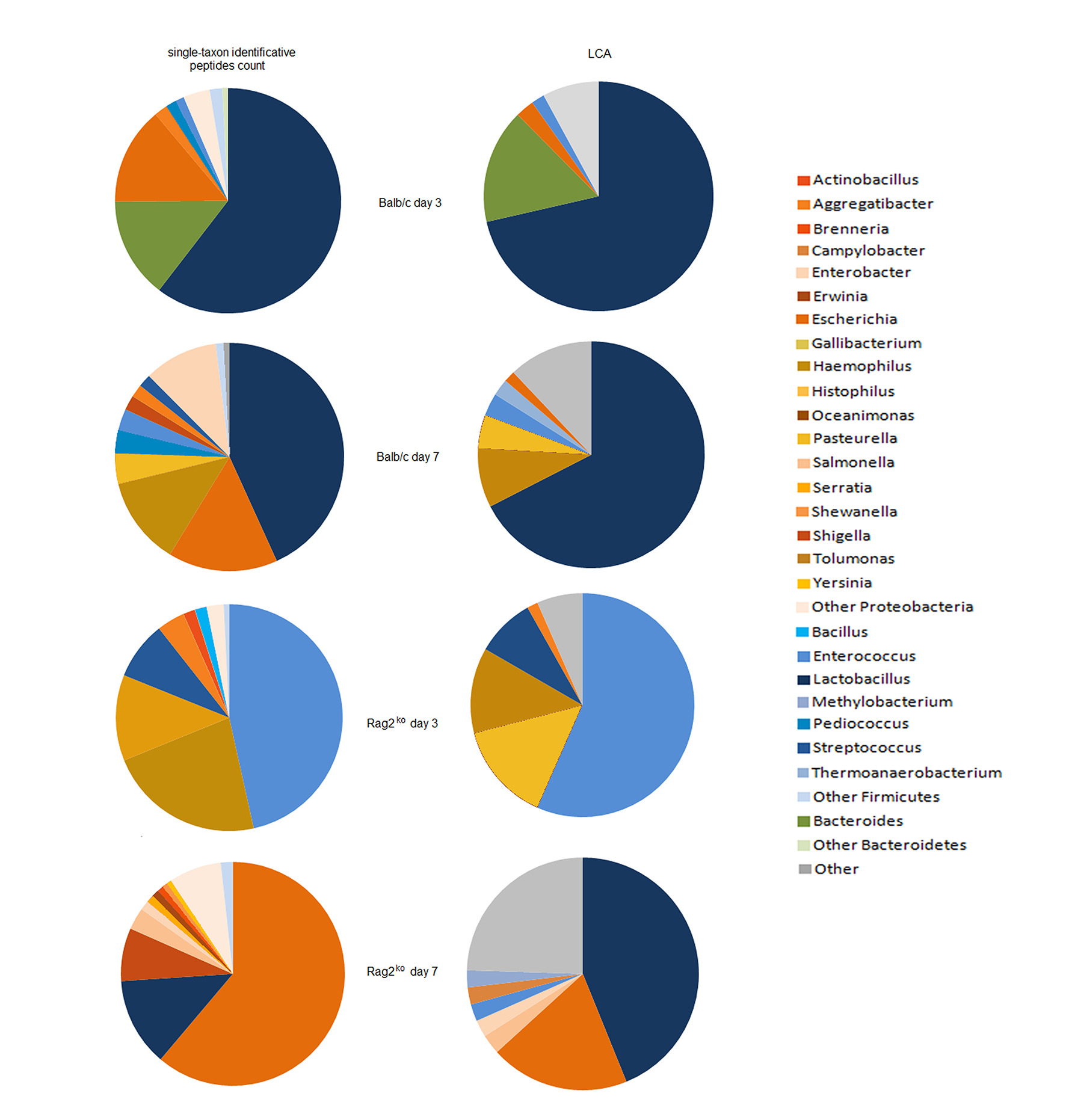

Supplement: FIGURE S2 — Comparison between workflow B and Lowest Common Ancestor analysis. When representing operational taxonomy unit (OTU) distribution at the genus level, the picture that came from the workflow B counting procedure and the Lowest Common Ancestor (LCA) algorithm was very similar or even remarkably different, due to the degree of ramification of the respective family. The a priori filtering of our workflow increased the number of taxon-specific peptides for genera sharing a large portion of the genome with others. [file Image_2.TIF]
